# Multiple recent HCAR2 structures demonstrate a highly dynamic ligand binding and G protein activation mode

**DOI:** 10.1038/s41467-024-49536-y

**Published:** 2024-06-25

**Authors:** Aslihan Shenol, Ricardo Tenente, Michael Lückmann, Thomas M. Frimurer, Thue W. Schwartz

**Affiliations:** Novo Nordisk Foundation Center for Basic Metabolic Research, Faculty of Health and Medical Sciences, University of Copenhagen, Copenhagen, Denmark

**Keywords:** Cryoelectron microscopy, X-ray crystallography, G protein-coupled receptors, Drug discovery

## Abstract

A surprisingly clear picture of the allosteric mechanism connecting G protein-coupled receptor agonists with G protein binding—and back – is revealed by a puzzle of thirty novel 3D structures of the hydroxycarboxylic acid receptor 2 (HCAR2) in complex with eight different orthosteric and a single allosteric agonist. HCAR2 is a sensor of β-hydroxybutyrate, niacin and certain anti-inflammatory drugs. Surprisingly, agonists with and without on-target side effects bound very similarly and in a completely occluded orthosteric binding site. Thus, despite the many structures we are still left with a pertinent need to understand the molecular dynamics of this and similar systems.

## Abstract (web summary)

Hydroxycarboxylic acid receptor 2 (HCAR2) is an attractive drug target to treat inflammation. Here, Shenol et al. discuss recent structural findings that elucidate HCAR2 interactions with multiple orthosteric and an allosteric ligand and with G proteins, providing insight into signaling through this receptor.

## Introduction

HCAR2 is the receptor target through which the important ketone body, β-hydroxybutyrate (BHB), and the active metabolite of the multiple sclerosis drug dimethyl-fumarate mediate their anti-inflammatory actions^1,2^. HCAR2 is also responsible for the important flushing side-effect of niacin^3^, which is used for the treatment of dyslipidemia (acting through an HCAR2-independent mechanism^4^). At the beginning of 2023, no structures of any HCAR receptor were available. But over the last year, 2 crystal structures and a total of 28 cryo-EM structures of HCAR2 in both inactive and active conformations, both without any ligands bound or in complexes with eight different endogenous and synthetic HCAR2 agonists, as well as an allosteric agonist and positive allosteric modulator (ago-PAM), have been published (Table 1). In fact, the HCAR2 structures constituted about one-third of all family A GPCR structures deposited in 2023^5^.

**Table 1:** The 30 3D structures of HCAR2 published in 2023

Aricle—title	Submission date	Acceptance date	PDB#	Type	Resolution	G protein	Ligand
Yang, Y., Kang, H.J., Gao, R. et al. Structural insights into the human niacin receptor HCA2-Gi signaling complex. Nat Commun 14, 1692 (2023).	21.09.2022	06.03.2023	7ZL9 7ZLY 7XK2	X-ray X-ray Cryo-EM	2.7 Å 3.3-2.5 Å 3.1 Å	- - Gi	None (inactive) Apo (inactive) MK6892
Zhao, Ch. Wang, H., Liu Y. et al. Biased allosteric activation of ketone body receptor HCAR2 surprises inflammaQon. MolCell, Vol.83, 17 (2023)	22.02.2023	28.07.2023	8JHY 8JII 8JIL 8JIM	Cryo-EM Cryo-EM Cryo-EM Cryo-EM	2.87 Å 3.17 Å 3.05 Å 2.98 Å	Gi Gi Gi Gi	Comp9n Comp9n + Niacin Niacin MMF
Suzuki, S., Tanaka, K., Nishikawa, K. et al. Structural basis of hydroxycarboxylic acid receptor signaling mechanisms through ligand binding. Nat Commun 14, 5899 (2023).	28.02.2023	12.09.2023	8IHB 8IHF 8IHH 8IHI	Cryo-EM Cryo-EM Cryo-EM Cryo-EM	2.9 Å 3.0 Å 3.1 Å 3.2 Å	Gi Gi Gi Gi	GSK256073 MK6892 LUF6283 Acifran
Cheng, L., Sun, S., Wang, H. et al. Orthosteric ligand selectivity and allosteric probe dependence at Hydroxycarboxylic acid receptor HCAR2. Sig Transduct Target Ther 8, 364 (2023).	28.02.2023	21.08.2023	8JZ7	Cryo-EM	2.60 Å	Gi	MK6892
Pan, X., Ye, F., Ning, P. et al. Structural insights into ligand recognition and selectivity of the human hydroxycarboxylic acid receptor HCAR2. *Cell Discov* **9**, 118 (2023)	11.03.2023	28.11.2023	8IJ3 8IJA 8IJB 8IJD	Cryo-EM Cryo-EM Cryo-EM Cryo-EM	3.28 Å 2.69 Å 3.23 Å 3.25 Å	Gi Gi Gi Gi	Apo-active! Niacin Acipimox MK-6892
Park, JH., Kawakami, K., Ishimoto, N. et al. Structural basis for ligand recognition and signaling of hydroxy-carboxylic acid receptor 2. Nat Commun 14, 7150 (2023).	17.03.2023	19.10.2023	8H2G 8K5C 8K5D	Cryo-EM Cryo-EM Cryo-EM	3.01 Å 3.13 Å 3.74 Å	Gi Gi Gi	Niacin Acipimox GSK256073
Zhu, Sh. et al. Molecular recognitioon of niacin and lipid-lowering drugs by the human hydroxycarboxylic acid receptor 2. Cell Reports, Vol.42, 11 (2023)	31.03.2023	24.10.2023	8J6L 8J6I 8J6J	Cryo-EM Cryo-EM Cryo-EM	3.05 Å 2.92 Å 2.80 Å	Gi Gi Gi	Niacin MK6892 GSK256073
Mao, C., Gao, M., Zang, SK. et al. Orthosteric and allosteric modulation of human HCAR2 signaling complex. Nat Commun 14, 7620 (2023).	23.05.2023	12.11.2023	8J6Q 8J6P 8J6R	Cryo-EM Cryo-EM Cryo-EM	2.60 Å 2.55 Å 2.76 Å	Gi Gi Gi	Comp9n +BHB comp9n + Niacin MK6892
Yadav, M.K., Sarma, P., Maharana, J. et al. Structure guided engineering of biased-agonism in the human niacin receptor via single amino acid substitution. Nat Commun 15, 1939 (2024).	31.07.2023	18.02.2024	8IY9 8JER 8IYW 8IYH 8JHN	Cryo-EM Cryo-EM Cryo-EM Cryo-EM Cryo-EM	3.37 Å 3.45 Å 3.45 Å 3.3 Å 3.75 Å	Gi Gi Gi Gi Gi	Niacin Acipimox GSK256073 MK6892 MMF

**Fig. 1:**
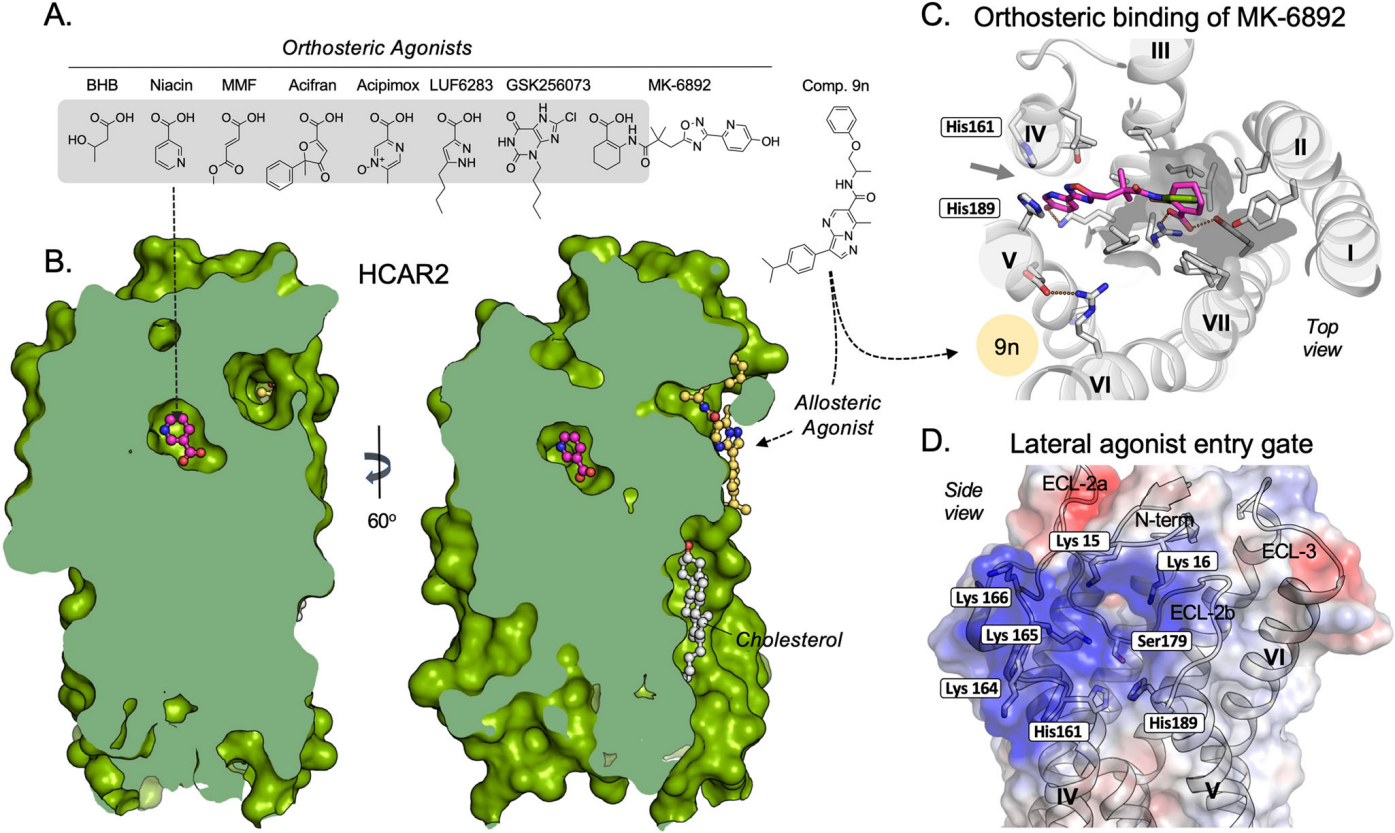
Binding modes for orthosteric and allosteric agonist (Ago-PAM) in HCAR2. A 2D structures of the HCAR2 agonists for which cryo-EM structures in complex with HCAR2 are available (see Table 1). **A** 2D structures of the HCAR2 agonists for which cryo-EM structures in complex with HCAR2 are available (see Table 1). The eight orthosteric agonists: b-hydroxy butyrate (BHB), Niacin, monomethyl fumarate (MMF), Acifran, Acipimox, LUF6283, GSK256073, and MK6892; and the ago-PAM, comp 9n. Grey shade—covers part of the agonists bound in the orthosteric pocket. **B** Orthosteric vs. allosteric agonist binding sites in HCAR2**—**binding of niacin (magenta) in the totally secluded orthosteric site at the center of HCAR2 versus concomitant binding of comp 9n (yellow) in the allosteric site/ grove between TM-V and -VI facing the lipid bilayer (PDB 8JII). In several HCAR2 structures cholesterol (grey) was bound in a site corresponding to the ‘inner leaflet agonist binding site’ described in FFAR1 between the intracellular segments of TM-IV and V^22,56^. **C** Top view of the orthosteric binding site with MK6892 (magenta) being anchored by an ionic lock between its carboxylate and Arg111 (located below) in the central orthosteric pocket and extending out between TM-IV and -V almost reaching the lipid bilayer below His161 and His189 in TM-IV and -V, respectively. The overlap of the benzoic acid moiety with Niacin (Green) in the central orthosteric pocket (grey shadow) is highlighted. The proposed lateral entry gate between TM-IV and -V is indicated by an arrow. The ionic lock between Glu190^5.40^ (TM-V) and Arg251^6.55^ (TM-VI) was observed in all active conformations and the binding site for comp 9n is also indicated. **D** Sideview of the proposed agonist entry gate to the orthosteric pocket between TM-IV and -V with electrostatic potential highlighted. The cluster of positively charged (blue) Lys residues at the top of TM-IV and at the end of the b-hairpin of the N-terminal segments are indicated above the important His161^4.59^ and His189^5.39^. Inside the opening of the entry gate is seen ECL-2, which the small agonists must pass under to reach the orthosteric site, with Ser179 which makes a stabilizing hydrogen bond to His189^5.39^ when it moves in to ‘close the door’ of the entry path. The PDB:8JIL structure has been chosen to illustrate the entry gate because it is one of the few structures where sidechains have been identified in the exterior part of HCAR2, in this case, the N-terminal segment.

**Fig. 2:**
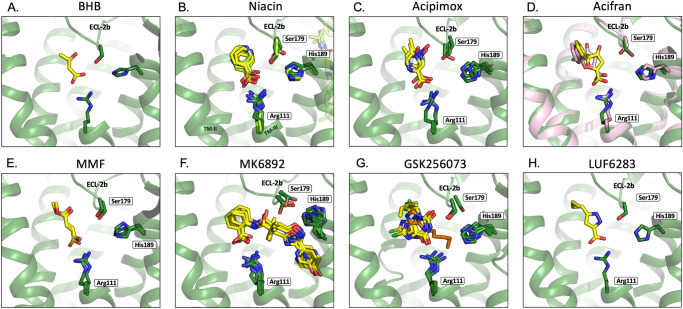
Binding poses for the eight different HCAR2 agonists binding in the orthosteric site. For all compounds a side view looking in between TM-II and -III are shown with the agonist-anchoring Arg111^3.36^, Ser179^ECL2b^ and the proposed entry/exit path-gating His189^5.39^ shown in stick models—note how the OH of Ser179^ECL2b^ alternates between interacting with the agonist and with His189^5.39^. Only helices of a single HCAR2 structure are shown for simplicity and part of TM-III is not displayed to view the residues behind better. **A** The single structure of BHB in HCAR2 in complex with the ago-PAM, cmpd9n, partly visible in the back to the right (PDB:8J6Q). **B** The seven structures of niacin in the two different rotamer poses, five structures with only niacin (PDB: 8JIL, 8IJA, 8H2G, 8J6L, and 8IY9) and two in complex with cmpd9n (PDB: 8JII, and 8J6P). **C** The three structures of Acipimox (PDB: 8IJB, 8K5C, and 8JER). **D** The single structures of Acifran in HCAR2 in yellow (PDB:8IHI) and the single structure of Acifran in HCAR3 in light magenta (PDB:8IHJ)—note the similar positioning of the three key residues and the similar pose of the acifran ligand in the two different receptors. **E** the two structures of MMF (PDB: 8JIM,8JNH); **F** the six structures of MK6892 (PDB: 7XK2, 8JZ7, 8IJD, 86JI, 8J6R, 8IYH)—note the different position of in particular His189^5.39^ which here is directly interacting with the ligand. **G** the four structures of GSK256073 of which three are in the same pose—in yellow (PDB: 8IHB, 8K5D, and 8IYW) and one in a different pose—in orange (PDB:8J6J), **H** the single structure of LUF6283 (PDB:8IHH).

**Fig. 3:**
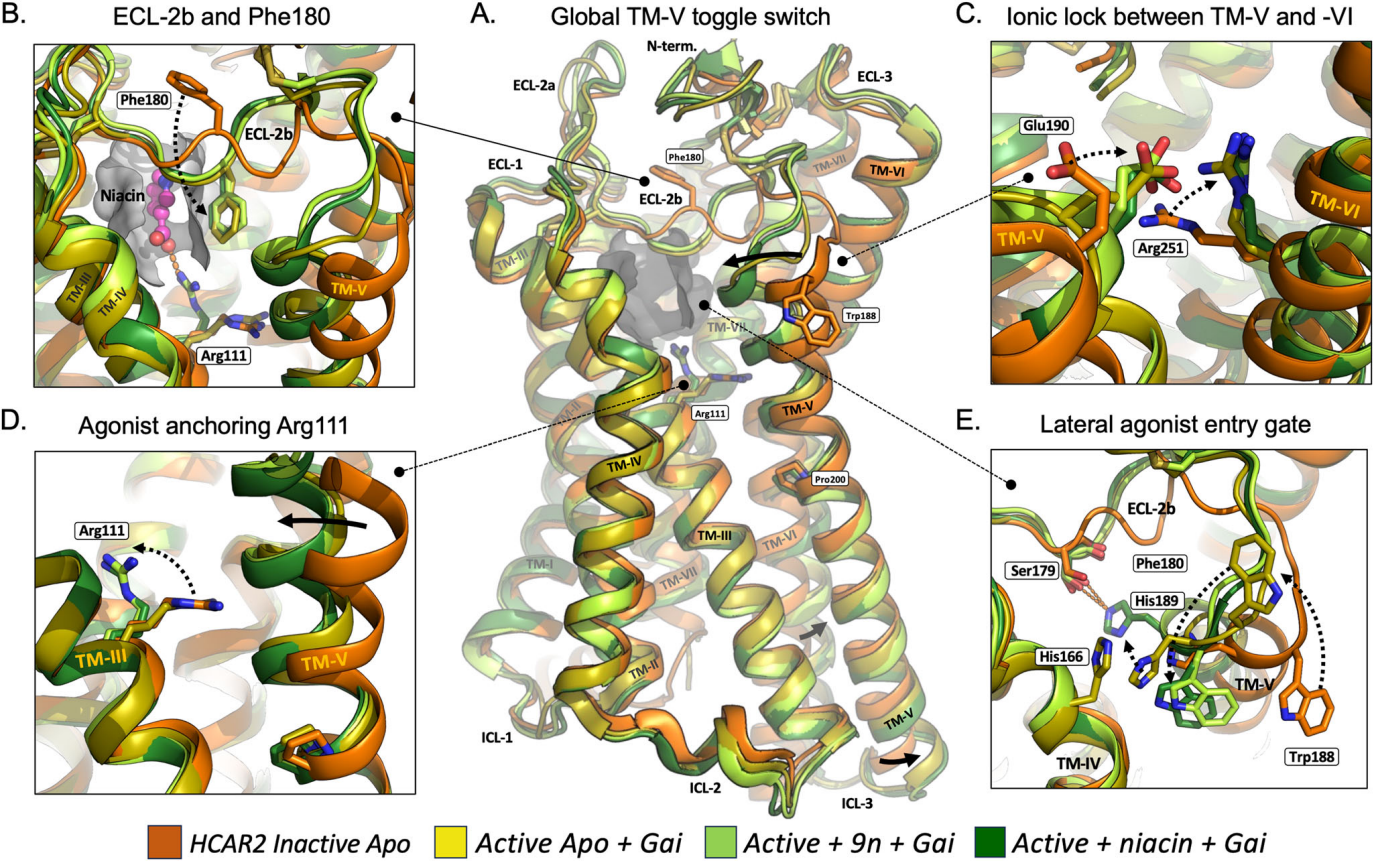
Activation Mechanism for HCAR2. Conformational changes from (1) inactive apo-form—PDB:7ZL9 (orange/brown), to (2) active apo-form in complex with Gai—PDB: 8IJ3 (yellow), to (3) active form in complex with the ago-PAM, comp 9n plus Gai—PDB:8JHY (light green), to (4) active form in complex with the orthosteric agonist niacin plus Gai—PDB 8JII (dark green), of which the latter corresponds closely to all the other agonist bound structures of HCAR2 (see Table 1). Only the receptor structures and no part of Gai structures or fusion proteins are shown. **A** Global toggle switch activation mechanism of HCAR2. A side view of the backbone structures of the four different forms of HCAR2 with an indication of the inner surface of the orthosteric binding pocket (grey shadow) and a few residues corresponding to s (**B–E**). Arrows indicate the toggle-switch movement of TM-V, i.e. outward movement of the intracellular pole and inward movement of the extracellular pole going from inactive to active conformation. The conserved, central, pivot point Pro200 in TM-V is indicated. **B** Conformational changes in ECL-2b and Phe180, which shifts ~10 Å down into the orthosteric pocket (grey shadow) from inactive to active conformation. Arg111^3.36^ and niacin are also indicated. View in through the entry/exit path between TM-IV and -V. **C** Formation of the ionic lock between the top of TM-V and -VI, i.e. between Glu190^5.40^ and Arg251^6.55^ in all three active forms of HCAR2 in complex with Gai. Inward view between TM-V and -VI. **D** Rotation of Arg 111^3.32^. Zoom in on Arg111^3.36^, which is only rotated up in the orthosteric pocket in the presence of either the receptor in complex with the orthosteric agonists (in this case niacin) or the ago-Pam, cmpd 9n, but not in the active apo-form in complex with only Gai. **E** Lateral Entry/Exit gate between TM-IV and -V. Top/inward view of TM-V and ECL-2b looking towards TM-VI. Note the unwinding of the first helical turn of TM-V from the inactive (orange) structure to the active Gai- bound apo-form (yellow) resulting in the distinct, different upward position of Trp188^5.38^ (long black hatched arrow) while the key gating residue His189^5.39^ moves inward to interact with His161^4.59^ in TM-IV. In the active structure in complex with the ago-PAM 9n (light green) and active niacin-bound (dark green) structures His189^5.39^ (totally overlapping) has moved further inward (black hatched arrow) to close the entry-exit gate and is stabilized here through H-bond formation with Ser179 in ECL-2. In these two agonist-bound structures the first helical turn of TM-V has reformed and Trp188^5.38^ has moved down again behind His189^5.39^.

Around 20 years ago, HCAR2—at that time known as GPR109A, PUMA-G, or HM74A—was deorphanized as the receptor for niacin by Offermanns and coworkers^6–8^ who also at an early stage identified a particular residue, Arg111^3.36^ as the key anchor point for the carboxylate of niacin. This residue is located one helical turn below the position of the canonical interaction point for monoamines in e.g. adrenergic receptors, Asp^3.32^ ^9^ The recent tsunami of HCAR2 structures all confirm that notion but, particularly when considered together, these structures also tell us surprising new stories about an occluded deep orthosteric binding site, with a dynamic entry/exit gate, and how an allosteric modulator works by gating this entry/exit path (Fig. 1). But, most importantly, the multiple structures (Fig. 2) with and without agonists and G protein clearly display the global two-way, allosteric mechanism connecting agonist and G protein binding in the receptor (Fig. 3).

## The first HCAR2 structures

In March 2023 Yang and coworkers published two X-ray structures of a mutated, thermostabilized HCAR2 locked in an inactive apo-form plus the first cryo-EM structure of HCAR2 in complex with Gi and the highly potent and selective, synthetic agonist MK6892 (Table 1)^10^. These structures revealed that the side chain of Arg111^3.36^ undergoes a major rotation from the inactive to the active conformation, which results in a 6.5 Å movement of its terminal guanidinium moiety up toward the center of the orthosteric pocket where it anchors the agonist through its essential carboxylate (Figs. 1C, 2, and 3A and 3D). Importantly, activation of HCAR2 was found to be associated with a vertical, ‘global toggle switch movement’ of TM-V around its central Pro200^5.50^ ^11^ in which its extracellular segment moves 5 Å inward to shrink the central pocket around the agonist headgroup while its intracellular segment moves 2 Å outward accompanying the canonical outward movement of TM-VI to encompass helix-5 of Gαi^10^ (Fig. 3A). The outward movement of the intracellular segment of TM-VI is apparently rather small in HCAR2, which likely is due to a constraint induced by the fusion protein replacing ICL-3 in the X-ray structure of the inactive apo-form of HCAR2. In Alpha-fold models of the inactive HCAR2 apoprotein without G protein, the intracellular pole of TM-VI is in general positioned further towards the center of the receptor. The headgroup of MK6892 makes several additional H-bond and hydrophobic interactions with residues in TM-II, -III, -VII, and ECL-2 in the central orthosteric pocket, and from here the relatively large agonist projects out between TM-IV and -V (Figs. 1C, and 2F).

## The subsequent flush of HCAR2 structures with multiple agonists

In the weeks before and after the publication of Yang et al. eight other reports were submitted each presenting one to five HCAR2 cryo-EM structures^12–19^ (Table 1). In six of these papers, the binding pose of MK6892 was confirmed^13–15,17–19^ and in five papers the binding pose of niacin in the central orthosteric pocket between TM-II,-III, -V, and -VII covered by ECL-2b was confirmed (Fig. 2B)^12,13,16,18,19^. This pocket was—as expected—shown also to be the binding site for other small synthetic agonists: Acifran (Fig. 2B)^17^, Acipimox (Fig. 2C)^16,18,19^, LUF6283 (Fig. 2H)^17^, and the similar size, non-carboxylate GSK256073 agonist (Figs. 1A and 2G)^13,16,17,19^. The endogenous ketone body, BHB, also binds to the orthosteric pocket (Fig. 2A)^14^ as did monomethyl-fumarate (MMF) (Fig. 2E), the HCAR2-activating metabolite of the multiple sclerosis drug, dimethyl-fumarate^12,19^.

Collectively, this ensemble of structures reveals the structural basis for the unique ability of the orthosteric pocket of HCAR2 to bind a multitude of chemically different small molecule ligands that all act as HCAR2 agonists (Figs. 1A and 2). All but one of these orthosteric agonists interact closely with the upward rotated Arg111^3.36^ via a salt bridge to their spearhead carboxylate group, while GSK256073 binds to Arg111 via one of its ketone groups (Fig. 2). In addition, the small agonists and the cyclohexene moiety of MK6892 make different types of hydrophobic, Van der Waals, aromatic, and hydrogen-bond interactions with side chains of Ser179, Met103, Leu104, Leu107, Ala108, Leu83, Trp91, Tyr87, Phe277, Leu280, Tyr284 and Phe180 in the orthosteric pocket, in line with their different chemical properties and binding pose within the pocket. The five different structures in complex with the relatively large molecule MK6892 are highly similar (10)^13–15,17–19^ (Fig. 2F), which is also the case for the six structures in complex with niacin and the two with MMF, although niacin is proposed to bind in two different conformations (Fig. 2B)^12,13,16,18,19^. However, for GSK356073, two very different poses were reported where either one or the other of its ketone groups are interacting with Arg111^3.36^, which positions the 8-chloro and the pentyl group of GSK356073 in two very different places in the pocket (Fig. 2G)^13,16,17,19^. In this connection, it should be recalled that the resolution of these cryo-EM structures is moderate (2.85–3.74 Å), which means that details around the ligand placement can be subject to bias in either the selection of models during the ligand fitting protocol or in the selection of a starting configuration. It is also possible that in this case, GSK356073 in reality may adopt each of the proposed poses in a substantial amount of its resident time.

Importantly, a common feature for all the orthosteric agonists is that despite their very different structures and different, specific interaction points, they all stabilize the same overall active, global toggle-switch conformation of TM-V and thereby indirectly also TM-VI—without directly interacting with residues in TM-VI as initially described for MK6892^10^.

## Agonists likely enter the deep orthosteric pocket through a lateral entry/exit path

The small orthosteric binding pocket of HCAR2 is located deeply buried below a tightly packed ‘lid’ composed of the folded structure of ECL-2 overlayed with the β-hairpin structure of the N-terminal segment (Figs. 1B and 3A)^10,12–20^. This lid is tied down to the top of the seven helical bundles by not only the canonical disulfide bridge from the top of TM-III to the middle of ECL2, but by two additional disulfide bridges from two neighboring Cys residues in the N-terminus to ECL2 and the top of TM-VII, respectively (Figs. 1B and 3A). In contrast, the orthosteric binding pocket in, for example, monoamine receptors, is located similarly within the 7TM helical bundle but, importantly one helical turn ‘higher up’ and with rather direct access from the extracellular aqueous phase which only requires relatively minor conformational changes such as adjustment of sidechains for the ligand to bind^21^. Binding of agonists to the orthosteric pocket in HCAR2 appears to be impossible from the top without major conformational changes (Figs. 1B and 3A). The Fujiyoshi group hypothesized that the electronegative HCAR2 agonists instead enter laterally through a highly electropositive opening between the extracellular segments of TM-IV and -V and ‘the lid’^17^. Here, the long-range electrostatic force of a cluster of positively charged lysine residues at the top of TM-IV and the start of ECL-2a, Lys164^4.62^, Lys 165^4.63,^ and Lys166^4.64^, in combination with Lys15 and Lys16 at the tip of the N-terminal β-hairpin motif will likely attract and ‘catch’ the agonist, which subsequently can enter the orthosteric pocket at the gap between the two lysine clusters just above the also likely positively charged His161^4.59^ and His189^5.39^ ^17^ (Fig. 1D). It should be noted, however, that the structural details and positioning and annotation of residues in the -hairpin of the N-terminus varies considerably between the different structures, probably due to the general low resolution of these structures in the exterior part of the receptors. The ‘tail’ of the relatively long MK6892 agonist is pointing back to a position just inside, below His161^4.59^ and His189^5.39^—i.e. below its likely entry site^10,12–15,18^. (Fig. 1C). A detailed molecular dynamics analysis of how a similar epitope of—in that case—five arginine residues catch and binds the electronegative dicarboxylic acid metabolite, succinate in the SUCNR1 receptor has just been reported^20^. In HCAR2, the concept of the lateral agonist entry path was supported by mutational analysis of the likely main gating residues, including His189^5.39^ as well as by molecular dynamics (MD) simulations trying to study ligand exit^17^.

Park and coworkers also noted the cluster of lysine residues at the interface between TM-IV and ECL-2a as a possible initial attachment site for the electronegative ligands, but instead favored His9 and Arg22^1.27^ in the N-terminal segment/TM-I at the top of ‘the lid’ as being involved in initial agonist attachment and entry also based on mutagenesis and MD simulations studying ligand interactions from the exterior^16^. Thus, the entry/exit path for the different agonists to the occluded orthosteric binding pocket in HCAR2 still awaits to be identified, e.g. through dedicated MD simulations.

Nevertheless, lateral entry of ligands from the lipid bilayer has been described not only in many GPCR sensors for lipid messengers but also in e.g. the MT1 melatonin receptor^22^. In that case, the neurohormone melatonin, a serotonin metabolite, enters a channel leading to the orthosteric binding pocket through a gate located at an almost identical place i.e. between TM-IV and -V. Moreover, in the odorant receptor OR51E2, where the short-chain fatty acid propionate binds in a similarly secluded, central orthosteric binding site, the agonist also appears to enter through a lateral highly dynamic entry gate, which however is located between TM-V and -VI^23^. Importantly, in OR51E2 the inward movement of the extracellular segment of TM-VI instead of TM-V as in HCAR2, is part of a similar even more clear global allosteric mechanism connecting agonist and G protein binding through a vertical toggle switch movement of in that case TM-VI^23^.

## The lateral entry path is gated by a dynamic gate

The short MD simulations that were performed to try to study ligand exit in HCAR2 demonstrated the dynamic nature of the presumed gate between TM-IV and -V, i.e. when the stabilizing small agonist was initially removed from the deep orthosteric pocket^17^. When comparing the four main types of structures (Fig. 3), it is evident that His189^5.39^ plays a particularly important role as a dynamic gating residue in the agonist entry/exit path. Thus, although the Cα of His189^5.39^ has moved toward the center of the receptor in the ligand-free active conformation with Gαi bound as compared to the inactive form, and the sidechain in this position interacts with His166 in TM-IV (yellow structures in Fig. 3E); His189^5.39^ moves further in and thereby blocks the entry/exit path when an agonist is bound In the deep orthosteric site (arrow from yellow to dark green in Fig. 3E). His189^5.39^ is stabilized in this blocking position mainly by H-bond formation with the sidechain hydroxyl and/or backbone carbonyl of Ser179^ECL2^ (Fig. 3E).

However, although the sidechain of Ser179 in several structures points to the imidazole ring of His189^5.39^, it is in other structures modeled to instead interact with the agonist (Fig. 2A–F). Nonetheless, when Ser179^ECL2^ was mutated to an Ala, it did not—as otherwise expected for an agonist-interacting residue—impair agonist function, but surprisingly instead strongly improved both the potency and the efficacy of e.g. the GSK256073 agonist^12,19^. The reason could be that hydrogen bond formation with Ser179^ECL2^ does not significantly contribute to the binding free energy of niacin but that substitution with alanine makes it easier for the ligand to get access to the orthosteric site. Importantly, in a native setting, the sidechain of Ser179^ECL2^ may simply alternate between these interactions. Moreover, the backbone carbonyl of Ser179^ECL2^ is in all structures position to form an H-bond to His189^5.39^ either directly or via a water molecule (Fig. 3E).

Thus, it appears that His189^5.39^ functions as a dynamic gate for the entry/exit path for the orthosteric agonists and is able to ‘close the door’ behind the bound agonist, i.e. prevents it from leaving the orthosteric site.

## Allosteric binding of an ago-PAM

In 2012 a class of HCAR2 ago-PAMs, i.e. allosteric agonists, which also act as positive allosteric modulators, e.g. niacin, were discovered by the Ijzerman group^24^. We now learn that the prototype of these substituted pyrazolopyrimidines, compound 9n, is in fact Gi-biased both as an allosteric agonist on its own and as an allosteric modulator, by only stimulating HCAR2-mediated Gi dissociation and cAMP inhibition and not β-arrestin recruitment^12,15^. Importantly, the four ccryo-EM structures of HCAR2/Gαi in complex with either 9 n alone or together with BHB or niacin show that 9n binds in a lipid-facing groove between the extracellular segments of TM-V and -VI, i.e. ~14 Å away from the central orthosteric binding site on ‘the opposite side’ of TM-V^12,14^ (Fig. 1B). The binding mode and receptor interactions are almost indistinguishable for 9n whether it binds alone^12^, i.e. acts as an allosteric agonist, or whether it binds together with BHB or niacin and acts as an allosteric enhancer^12,14^. Interestingly, although it is argued by the authors that there are a couple of small changes in the positioning of side chains in the orthosteric pocket around niacin and that the volume of the orthosteric pocket is slightly smaller when 9n is bound in the allosteric site^12^, these differences do not convincingly explain how 9 n acts an allosteric enhancer.

## The allosteric modulator appears to function as a gatekeeper for the dynamic entry/exit gate

We favor an alternative mode of action where 9n instead acts as a PAM by controlling the dynamics of the “gate” residue His189^5.39^ of the orthosteric pocket (Fig. 3E). Interestingly, the allosteric agonist 9n binds between the outward-facing, extracellular segments of TM-V and -VI and thereby stabilizes these two helices in an active conformation which is very similar to that observed in the structures with small agonists bound in the orthosteric site, including the formation of the ionic lock between Glu190^5.40^ and Arg251^6.55^, which in fact are part of the actual binding site of 9n^12^ (Fig. 1C, and light vs. dark green structures in Fig. 3C).

Nevertheless, we would argue that 9n by itself and in complex with a small orthosteric agonist will stabilize a receptor conformation in which His189^5.39^ is held in the inward position partially blocking the entry/exit path for the orthosteric agonists and thereby—if it binds after the agonist—keeps the gate firmly closed behind e.g. niacin, which has passed His189^5.39^ on its way into the orthosteric site (Fig. 3E). Thus, it could be argued that 9n acts as a PAM for, e.g., niacin or BHB by slowing down their exit, i.e. their off-rate, and consequently prolonging their receptor occupancy and thereby action. An analogy to this mode of action would be how the PAM LY2119620 in the M2 muscarinic receptor (PDB ID: 4MQT) binds in an allosteric site located directly in the otherwise open entry/exit path for the orthosteric muscarinic agonist towards the aqueous, extracellular phase^25^. However compound 9nbinds far away from the proposed orthosteric agonist entry path, i.e. on the opposite side of TM-V, but nevertheless indirectly closes the entry/exit path off by changing the overall conformation of TM-V and – importantly—the location and interaction of the key gating residues (Fig. 2A and E). Interestingly, 9n only acts as an enhancer for the small orthosteric agonists and not for MK6892^14,15^ conceivably because binding of 9n prevents MK6892 binding due to the 9n-induced changes in the gate area (Fig. 3C), where the tail of MK6892 normally binds (Fig. 1C)^10,12–15,18^.

## Agonist and G protein binding are interconnected by a global allosteric selection mechanism

The availability of structures of the HCAR2 apo-protein in not only inactive^10^ but rather extraordinarily also in an active conformation, i.e. in complex with Gαi without any ligand^18^ (PDB ID 8IJ3), provides a unique insight into the allosteric mechanism linking the intracellular G protein binding site with the orthosteric binding site. Thus, the overall conformation of TM-V is almost identical in the HCAR2/Gαi apo-state structure to the one observed in all the many agonist complexes, i.e. locked in the active global toggle-switch conformation with the intracellular segments of TM-V and -VI moved slightly outwards to accommodate binding of the C-5 helix of the Gαi protein, and—importantly—with the extracellular segment of TM-V moved inward without any agonist bound ‘inside’ (Fig. 3A)^18^. The inward bend conformation of TM-V is stabilized by the salt bridge between Glu190^5.40^ and Arg 251^6.55^ (Fig. 3C). Interestingly, binding of Gαi also stabilizes the backbone conformation of ECL-2b from a position where the sidechain of Phe180^ECL2^ points upward and away to a conformation almost identical to that observed in the many agonist-bound structures where Phe180^ECL2^ instead points downwards into the orthosteric pocket (yellow vs. orange in Fig. 3B).

Interestingly, the binding of Gαi is not associated with rotation of the sidechain of the agonist anchoring Arg111^3.36^ as observed in all the agonist-bound receptors (Fig. 3D). This indicates that -Arg111^3.36^ is not per se part of the allosteric communication between G protein and agonist binding. However, the rotation of the sidechain of Arg111^3.36^ is observed in the ago-PAM bound receptor although this ligand binds far away from Arg111^3.36^ (light green, Fig. 3D). A potential explanation for this might be based on indirect PAM-mediated effects via residues further down TM-V, which Arg111^3.36^ interacts with in the inactive state.

Notably, the conformational changes occurring around the presumed main ligand entry/exit path from the inactive apo-form (Fig. 3E, orange) to the Gαi-bound apo-form (yellow) differ from those observed in the ago-PAM (green), and agonist bound (dark green) active forms. Thus, although TM-V has moved inward to a similar degree from the inactive to the three ‘active’ forms (Fig. 2A) the entry/exit path between TM-IV and -V is not as efficiently closed in the Gαi apo-form as in the agonist and ago-PAM bound forms (Fig. 3E). Although His189^5.39^ in the Gαi-apoform is positioned at the interhelical interphase where it interacts with His161^4.59^ in TM-IV (yellow residues in Fig. 3E), In fact, the first helical turn of TM-V appears to be partly unfolded in the Gαi-bound form as Trp188^5.38^ points up and away from the entry path (yellow, Fig. 3E), which could help agonist binding. In contrast, in the agonist-bound, active form the first helical turn of TM-V has formed and both the Cα carbon and the sidechain of His189^5.39^ have in all agonist-bound structures moved further in to close off the entry/exit path efficiently by interacting with the residues in ECL-2b—and Trp188^5.38^ has moved in behind (Fig. 3E).

The unique collection of diverse HCAR2 structures illustrates how agonist and G protein binding to the extracellular and intracellular domain of the receptor, respectively, stabilize the same overall active receptor conformation—in agreement with a Monod, Wymann, Changeux selection-type of allosteric mechanism connecting G protein with agonist binding—and back^11,26^. The fact that G protein binding does not stabilize the “active” conformation of Arg111^3.36^ underlines that this key agonist-anchoring residue apparently does not function as the first—or last—residue in a ‘propagating path’, induction-type of allosteric mechanism corresponding to some kind of internal ‘domino brick’-like line of residues connecting agonist binding with G-protein binding, which often is illustrated in publications of novel GPCR structures^27,28^.

Consequently, it could be argued that the main—or in fact the only effect—agonist binding has on the HCAR2 structure, which is not already mediated by Gαi protein binding, is the induced fit in respect of the ligand binding as such, i.e. stabilizing the rotated conformation of the anchoring Arg111^3.36^ and ‘closing of the door’ in the entry path to the orthosteric pocket (Fig. 3D, E). Otherwise, Gαi binding to the intracellular domain on its own stabilizes all the major conformational changes that characterize the active state of the extracellular receptor, i.e. conformational changes observed in the presence of Gαi plus the agonists.Importantly, inward movement of the extracellular pole of TM-V (yellow on top of the two greens in Fig. 3A) plus the downward movement of ECL-2 and Phe180 (Fig. 3B), and formation of the stabilizing ionic lock between TM-V and -VI (Fig. 3C).

The many recent structures of agonist and ago-PAM bound HCAR2 emphasize the original notion that there is no definitive, singular “active site” in GPCRs to which an agonist has to bind^11,29^. Agonists can bind anywhere in the receptor as long as it stabilizes an active conformation, which the receptor can adopt on its own and which – importantly—also is stabilized by the appropriate intracellular G protein^11^—a point which often is forgotten or underappreciated.

## HCAR2 as a drug target – from dyslipidemia and diabetes to inflammation

HCAR2 (known then as GPR109A)first came to the attention of the scientific community as a drug target. It was deorphanized as the receptor for vitamin B3, niacin, which since 1955 had been—and still is—used for the treatment of dyslipidemia^6–8^. Niacin and its analogs Acifran and Acipimox (Fig. 1A) have clear beneficial effects on circulating lipoproteins but are not very efficacious and can cause debilitating facial flushing as a side effect. Accordingly, after the deorphanization, all major pharmaceutical companies and many biotech companies started large campaigns to develop novel GPR109A agonists, hoping to obtain more efficacious dyslipidemia drugs with fewer side effects^30^. But at an early stage it was realized that flushing is in fact an on-target mechanism-based side effect, mediated by HCAR2 itself expressed on Langerhans cells (a type of immune cells in the skin that release prostaglandins causing local vasodilation)^3,31^. However, it was proposed that flushing- as opposed to the beneficial effects of HCAR2 activation- was associated with receptor internalization and activation of ERK1/2 MAP kinase signaling^30^. Based on similar observations focused on arrestin signaling, HCAR2 was in 2009 highlighted by Lefkowitz and coworkers as a prime example of how the development of a biased agonist which signals through G protein but devoid of arrestin signaling, would provide the beneficial effect on dyslipidemia without the flushing side effects^32^. The first example of such a drug candidate was MK-0354^33^, which since has been followed by discovery of many others including LUF6283, GSK256073, and MK6892 shown in Fig. 1A^34–36^. However, although strongly promoted, these synthetic ligands did not completely lack vasodilatory side effects but at best had a certain bias in that direction.

All of this was done under the assumption that HCAR2/GPR109A was in fact mediating the beneficial effect of niacin on dyslipidemia. However, HCAR2/GPR109A is mainly expressed in adipose tissue where it inhibits lipolysis and its functional connection to the production of lipoproteins in the liver remained unclear. Importantly, when fully efficacious HCAR2/GPR109A agonists were eventually tested in clinical trials and in HCAR2/GPR109A knockout animals it became clear that although the compounds suppressed lipolysis, i.e. lowered circulating free fatty acids, they did not deliver the expected niacin-like effects on circulating lipoproteins in patients^4^. Subsequently, this lack of effect on lipoprotein levels was also reported for GSK256073^37^. Thus, although this eliminated HCAR2/GPR109A as a drug target for the treatment of dyslipidemia, HCAR2 agonists do inhibit lipolysis, which potentially could be beneficial in e.g. diabetes. However, clinical trials with e.g. GSK256073 in diabetic patients demonstrated that the effect on lipolysis, unfortunately, disappears during prolonged treatment as it appears to be subject to the development of tolerance^38,39^.

Although niacin acts as an agonist on HCAR2 when given as a drug, the circulating concentrations of endogenous niacin made by e.g. the gut microbiota are under physiological conditions too low, and in 2005 it was shown that the endogenous ligand for HCAR2 in fact is the ketone body, BHB^1^ (Fig.1). This eventually led to the discovery that HCAR is responsible for the anti-inflammatory effects of not only BHB^40^ but also the multiple sclerosis drug dimethyl fumarate^2,41,42^.

Thus, although HCAR2 is no longer a valid drug target in dyslipidemia and likely not in diabetes either, the many HCAR2 ligands could potentially become useful as anti-inflammatory agents in the treatment of neuroinflammation and e.g. psoriasis^42,43^.

## What differentiates HCAR2 agonist with and without the flushing side effect?

In connection with the structural biology characterization of the binding modes for the different HCAR2 ligands (Fig. 1), many of the agonists were revisited concerning their signaling properties. Surprisingly, these studies appear to change our understanding of the suspected biased signaling of key ligands. Yang et al. now report that the high potency HCAR2 agonist, MK6892, which was reported to cause very limited flushing^32,33,36^ and was supposed to be devoid of arrestin signaling^32,33^, in fact signals through arrestin at a level similar to niacin^10^ This result was confirmed by Yadav et al.^19^. Moreover, another “non-flushing” HCAR2 agonist GSK256073^34^ is in fact even more potent and efficacious than niacin- not only in respect of G protein signaling but also in respect to arrestin recruitment^19^. These observations obviously cast strong doubt on the hypothesis that lack of arrestin signaling should be responsible for lack of flushing/ vasodilation and thereby invalidate the concept that G protein-biased HCAR2 agonists will be devoid of the flushing side effect^32,33^. However, a thorough, parallel signal transduction analysis of all these—and other—“flushing” and “non-flushing” HCAR2 agonists is required to fully understand whether or rather how these two classes of HCAR2 agonists are different from each other with respect to signaling, if at all.

When comparing the available 28 different HCAR2 structures with nine different agonists bound (including 5 very similar structures with each of the key ligands: niacin, MK6892, and GSK256073), there are, somewhat disappointingly, no obvious differences between the “non-flushing” and “flushing” agonist in respect of binding mode. Obviously, the relatively large MK6892 occupies a larger pocket compared to niacin. However, the other “non-flushing” agonists LUF6283 and GSK256073 occupy the same orthosteric pocket as do the “flushing” niacin, acifran, and acipimox agonists (Fig. 1). The different agonists do make different types of interactions within this pocket as discussed in the individual papers (Fig.2, Table 1), but how this is associated with causing flushing or not, is totally unclear. Importantly, the overall conformational change of the helical bundle is very similar to all the different agonists—as discussed above.

## Design of novel HCAR2 receptor ligands to target receptor dynamics?

Although itis mentioned in all nine papers that their respective new 3D structures will guide future drug discovery/development in the HCAR2 field, this is only done in general terms and no specific guidance is provided as to how specific features in these “frozen structures” actually will help and guide the drug discovery process^10,12–19^.

Importantly, it is becoming increasingly clear that the pharmacological properties of ligands are determined not only by their final binding pose but to a surprisingly large degree by dynamic interactions of the ligands with the receptor along their entry path^20,21,44,45^. In this connection, the HCAR2 structure with its rather complex agonist entry/exit path for the orthosteric site offers clear possibilities to target this either directly or indirectly. The clearly indirect mode through which the ago-PAM, compound 9n affects this dynamic entry path underlines this potential—as discussed above.

However, what is really needed at this stage are large-scale, thorough studies of the molecular dynamics of the HCAR2 receptor as such, including unbiased MD simulations and e.g. metadynamics simulation studies of binding and exit paths for the many different types of HCAR2 agonists. Moreover, molecular dynamics studies of G protein—and perhaps arrestin—binding performed by e.g. Gaussian accelerated MD simulations^46,47^ could potentially also provide important new information about the receptor dynamics. It has also become increasingly clear that agonists and antagonists of GPCRs can target intracellular pockets where such ligands directly stabilize the active conformation of receptor domains that interact with the G-protein or prevent these from being formed^48–50^. In this connection, it should be emphasized that ligands can successfully be designed to bind to dynamic, cryptic pockets, which e.g. only are open in the active receptor conformation to thereby function as allosteric agonists—as done successfully in e.g. the GPR40/FFAR1 receptor^51^.

## Structures and drugs for the other two HCAR receptors?

The deep knowledge about HCAR2 structural biology, which is now available, appears to have broken the ice with respect to HCAR receptors in general. Thus, in one of the HCAR2 papers, the first ccryo-EM structure of the 3-hydroxy octanoate receptor, HCAR3, in complex with Acifran was reported^16^. As expected, based on the very high degree of primary sequence identity ( ~ 95%) and partial ligand overlap, the 3D structures of HCAR2 and HCAR3 were also very similar. This includes the organization of the deep, occluded orthosteric pocket and the proposed ligand entry/exit path between TM-IV and -V and the almost identical binding pose of Acifran in both HCAR2 and HCAR3^16^ (Fig. 2D). The physiological role of HCAR3 is believed to overlap in many respects with HCAR2, but since HCAR3 exists only in humans and other primates, its pharmacological potential is difficult to pursue due to lack of suitable in vivo pharmacological / toxicological models. Nevertheless, high-potency synthetic HCAR3 agonists with high selectivity towards HCAR2 have actually been developed for human HCAR3^52^.

The third HCAR receptor, HCAR1, which is a sensor of lactate, is found in all mammalian species and is a very interesting potential drug target. Although like HCAR2 it is normally expressed mainly in adipose tissue and various immune cells, it is highly upregulated in essentially all types of solid tumors induced by the high lactate in the tumor microenvironment^53^. Because HCAR1 apparently is involved in both the control of cancer cells metabolism and defense mechanisms as well as in the control of anti-cancer immune cells^54^, HCAR1 antagonists could potentially be highly interesting as anti-cancer drugs. However, antagonists are not yet available for any of the three HCAR receptors. Still, the availability of the multiple HCAR2 and the first HCAR3 structures together with the use of high-quality AlphaFold models exploiting the knowledge from the cryo-EM structures should provide a good basis for the discovery of not only agonists but also antagonists for both the HCAR2 and HCAR1 receptors.

## Outlook

The main message from the recent wealth of structural information concerning the HCAR2 receptor is that we now need to focus on understanding the dynamics of the system to really understand how the receptor works. This is true for any GPCR or for that matter any protein in general. But, in the case of HCAR2 very large conformational changes of the receptor—including opening and closure of the entry gate—will be required for ligand binding as the orthosteric binding site is completely occluded. Future studies will likely have to include large-scale, long-duration MD simulations, accelerated MD simulations, and metadynamics analysis and the potential development of improved software forcefields. Potentially such studies could be combined with e.g. time-resolved cryo-EM studies as recently performed in the analysis of the conformational trajectory underlying G protein activation during its functional dissociation from a GPCR^55^. It will be particularly interesting to determine to what degree the receptor on its own makes the crucial conformational changes and how these will be stabilized by binding of the G protein alone—providing constitutive signaling—and by the agonist alone and in combination with G protein. Understanding this dynamic interplay would provide better structural templates for compound screening to guide the design of selective agonists and antagonists.

What we got today corresponds to the glossy pictures in front of the movie theater. We want to get in and see the real movie, even though we do have a pretty good idea about the plot from the key frames that we can see out there.
